# Determinants of multimorbidity of infectious diseases among under-five children in Bangladesh: role of community context

**DOI:** 10.1186/s12887-022-03217-1

**Published:** 2022-03-28

**Authors:** Rashmi Rashmi, Ronak Paul

**Affiliations:** 1 Department of Population and Development, International Institute for Population Sciences, Mumbai, 400088 Maharashtra India; 2 Department of Public Health and Mortality Studies, International Institute for Population Sciences, Mumbai, 400088 Maharashtra India

**Keywords:** Comorbidity, Respiratory infection, Diarrhoea, Communicable diseases, Community environment, Bangladesh Demographic and Health Survey

## Abstract

**Background:**

The presence of more than one morbid condition among children has become a global public health concern. Studies carried out in Bangladesh have primarily focused on diarrhoea and acute respiratory tract infections independently without considering their co-occurrence effect. The present study examines the multimorbid conditions of infectious diseases in under-five Bangladeshi children. It explores multimorbidity determinants and the role of community context, which are often overlooked in previous literature.

**Methods:**

Utilizing the most recent Demographic and Health Survey of Bangladesh (2017–18), we used mixed-effects random-intercept Poisson regression models to understand the determinants of multimorbidity of infectious diseases in under-five Bangladeshi children considering the community-level characteristics.

**Results:**

The present study found that 28% of the children experienced multimorbidity two weeks prior to the survey. Community-level variability across all the statistical models was statistically significant at the 5% level. On average, the incidence rate of multimorbidity was 1.34 times higher among children from high-risk communities than children from low-risk communities. Moreover, children residing in rural areas and other urban areas involved 1.29 [CI: 1.11, 1.51] and 1.28 [CI: 1.11, 1.47] times greater risk of multimorbidity respectively compared to children from city corporations. Additionally, the multimorbidity incidence was 1.16 times [CI: 1.03, 1.30] higher among children from high-altitude communities than children living in low-altitude communities.

**Conclusion:**

The significant effect of public handwashing places suggests community-based interventions among individuals to learn hygiene habits among themselves, thus, the severity of coexistence nature of infectious diseases. A higher incidence of coexistence of such infectious diseases in the poor and semi-urban populace further recommends a targeted awareness of a clean environment and primary healthcare programmes.

## Background

According to a United Nations (UN) report, the last few decades have seen a remarkable improvement in women and child’s health and survival status [1]. Today, more children are surviving, but they are suffering from two or more ailments. [1]. Ample evidence shows that residing with multimorbidity (i.e., a complex state with few dominant patterns even among those with two or three conditions) has grown significantly in younger age populations [2]. The term multimorbidity is often used interchangeably with comorbidity. However, the concept of multimorbidity is distinct from comorbidity, as multimorbidity indicates that no single condition holds priority over any of the co-occurring conditions from the perspective of the patient and the health care professional [3]. Such occurrences are either simultaneous (occurring at the same time) or sequential (occurrence of one lead to the occurrence of the other). For instance, a study shows that the epidemiology of diarrhoea and pneumonia may overlap in under-five children as they may co-occur due to shared risk factors or under a vicious cycle [4, 5].

Though the world is seeing a shift in the disease burden, from infectious to non-communicable diseases [6], infectious diseases remain a higher cause of death among children under five years of age [6, 7]. The presence of more than one infectious disease in a child can endanger future survival and wellbeing [5]. The sudden emergence of endemic and epidemic (like coronavirus disease in 2019) can further worsen the situation [2, 8, 9]. So, together multimorbidity and infectious diseases in children is an urgent public health concern.

According to the World Health Organization, diarrhoea and lower respiratory infections ranked in the top ten causes of death, especially in young children [10]. Despite being preventable and treatable, common infectious diseases like diarrhoea, malaria, pneumonia are alone responsible for 29% of under-five deaths globally in 2018 [6]. About 4,80,000 children under five years of age lost their lives from diarrhoea in 2017, mostly from regions of South Asia and sub-Saharan Africa [6]. One of the South Asian countries, Bangladesh, has higher early-life mortality than the estimated target of Sustainable Development Goals [11, 12]. Diarrhoea and respiratory tract infections, including fever, cough, and breathing problems, are common among Bangladeshi children [13]. Studies have associated diarrhoea and respiratory tract infections with the exposure, household environment, and demographic characteristics of children [14–17]. Extant evidence shows that the risk of diarrhoea and acute respiratory infection was common in younger ages [18, 19]. Numerous studies from developing countries have also shown a significant effect of improved sanitation and water sources on the incidence of childhood diarrhoea [19]. One study from Bangladesh has shown that smoking habits among family members, location of the kitchen, and cooking fuels play an important role in acute respiratory infection incidence [20]. Studies have also shown that urban and male children were less likely to experience these diseases due to better food and health care facilities [21]. A UNICEF report shows that infectious diseases are more concentrated in the poorest regions and indicated that disparity in the primary healthcare services at community level were responsible for the same [6]. The geographical location, education and socioeconomic status of the community may play an essential role in predicting the presence of infectious diseases in children.

So far, knowledge of these determinants has helped Bangladesh bring several vertical programs focusing on the issue of child morbidity. However, over time, with the changing nature of diseases (in the form of multimorbidity as children are no longer battling one disease at a time), separate interventions for specific morbidities are often questioned. Thus, it becomes crucial to track the multimorbid face of infectious diseases in children, and their determinants, especially among the under-five children, who are more susceptible to infectious diseases than children of older age groups. Notably, there is a dearth of research on the determinants of multimorbidity of infectious diseases among Bangladeshi children. Additionally, the role of the community behind the occurrence of multimorbidity needs to be explored as children residing in the same community often share similar characteristics [22, 23]. This necessitates understanding the relationship of community-level contextual factors with the multimorbidity of infectious diseases in children. Therefore, this study explores the child, household and community-level determinants of multimorbidity of infectious diseases in under-five Bangladeshi children keeping in focus the role of the community behind the risk of multimorbidity. The current study hypothesizes that community characteristics play no role in multimorbidity among under-five children in Bangladesh.

## Methods

### Data source

The present study used the most recent Demographic and Health Survey of Bangladesh conducted during 2017–18 (referred to as BDHS 2017–18). The National Institute of Population Research and Training (NIPORT) conducted BDHS 2017–18 under the stewardship of the Ministry of Health and Family Welfare (MoHFW) of Bangladesh. This survey provided crucial information on maternal and child health, mortality and morbidity. Details regarding sample design, survey instruments, training and fieldwork, data collection and processing, and response rates are available in the BDHS 2017–18 report [11].

This study used the data for 8759 children under five years born to 7562 mothers aged 15–49 years across 672 communities in Bangladesh. However, we dropped the records of 361 children who were not alive during the survey and had no information regarding their morbidity status. Therefore, the analytical sample for this study is 8398 under-five children across 672 communities.

### Outcome variable

The outcome variable of multimorbidity status was constructed from the mother’s responses regarding their children’s morbidity status. BDHS 2017–18 collected information on whether the children had suffered from fever, cough, acute respiratory infections (ARI) and diarrhoea within two weeks before the interview. We combined these four variables into a count variable of morbidity status that contained five categories – children who did not suffer from any of the four morbidities (“no condition”), children who suffered from one (“single condition”), two, three and four conditions respectively. The advantage of this approach is that it allows us to consider the severity of the children’s infirmity in the sense that the greater the number of comorbid conditions, the more severe its effect on the health of children [13].

### Explanatory variables

Guided by extant research, we identified relevant factors associated with the occurrence of infectious morbidity among children [13, 14, 18]. Accordingly, we included relevant explanatory variables, conditional upon their availability in BDHS 2017–18. The child-level characteristics are – age in years (less than one, one, two, three, four) and gender (male, female). The parent-related characteristics are – number of under-five children under a mother (one, two, three or more), mother’s level of education (no formal education, upto primary, secondary and above), father’s level of education (no formal education, upto primary, secondary and above). The household-level factors are – sanitation condition (poor, average, good), the household had water treated before drinking (no, yes), type of handwashing place (private space, public place, no handwashing place), shares toilet with other households (not shared, shared by two households (HH), shared by three HH, shared by four and more HH), wealth quintile (poorest, poor, middle, rich, richest), the religion of household (Islam, Hinduism, others). Further, the season during the interview (summer, winter, monsoon) was also included as an explanatory variable. The community-level characteristics included were – type of community (city corporation, other urban areas, rural areas), altitude level of community (low, medium, high), socioeconomic status of community (low, medium, high), level of maternal education in the community (low, medium, high) and administrative division of community (Dhaka, Chittagong, Barisal, Khulna, Mymensingh, Rajshahi, Rangpur, Sylhet).

Guided by extant research, the household sanitation condition variable was constructed from three variables – type of source of drinking water, type of sanitation facility and the number of members per room in the household [24]. Respondents were asked about the source of household drinking water. As per prevalent standards, we recoded the source of household drinking water into two categories – “unimproved” (coded as 0) and “improved” (coded as 1) [25]. Similarly, we recoded the type of household toilet facility into – “unimproved” (coded as 0) and “improved” (coded as 1) [25]. Further, households with less than three members per room were coded as “1”, and those with three or more members were coded as “0”. After this, we added the three variables to obtain a household sanitation condition score. Households with a score of three, a score of two and a score less than two were categorized as having “good”, “average”, and “poor” sanitation conditions, respectively. Further, to avoid multicollinearity, we constructed a new wealth quintile variable after excluding household water source and toilet facility information. The modified wealth quintile variable was prepared using standard procedures that are documented elsewhere [26].

The community-level characteristics were constructed by aggregating the maternal and household-related information to the community level. This study refers to a primary sampling unit (PSU) as a community. The community’s altitude level was calculated using data on each community’s height (in metres) above sea level. We further classified the altitude level of the community as “low”, “medium”, and “high” based on three quintiles. The socioeconomic status of the community was defined as the proportion of “rich/richest” wealth quintile households in a community. Equivalently, the proportion of women aged 15–49 years with “secondary and above” education was used to determine the level of maternal education in the community. In addition, based on the terciles of each variable, we classified the community’s socioeconomic position and degree of maternal education as “low”, “medium”, and “high”.

### Statistical methods

We performed bivariate and multivariate analyses to realize the study objectives. The bivariate association of multimorbidity status with the child-level, parent-related, household-related and community-level explanatory variables were examined using the chi-square test for association. Multivariable analysis was performed by estimating mixed-effects random-intercept Poisson regression models, owing to the count nature of the multimorbidity status variable. The data was hierarchical, with children nested within households which in turn were nested within communities. Therefore, we estimated two-level random intercept Poisson models with communities at level-2 and children at level-1. We did not include a distinct household level in the multilevel models as the average number of children per household was relatively low (1.16 children per household).

We obtained the community-level Median Rate Ratios (MRR), which measures the variability in risk of multimorbidity among children across communities [27, 28]. The MRR is defined as the median of the relative change in the incidence rate of multimorbidity among all possible pairs of low-risk and high-risk communities. In pairs, the communities with high and low multimorbidity incidence are considered high-risk and low-risk communities, respectively. The MRR is always greater or equal to one, and the higher the MRR value, the greater is the heterogeneity in the risk of multimorbidity across communities. Further, the multivariate association of morbidity status of children with the explanatory variables was shown using incidence rate ratios (IRR). The IRR gives the risk of having morbidity compared to having no morbidity among children belonging to a particular category of an explanatory variable given the effect of all the other explanatory variables and the community-level variability remain constant [28]. We calculated three models for estimating the adjusted risk of multimorbidity – the null model is an empty model without any covariates, model-I includes all covariates excluding the community-level characteristics, Model-II is the full model that includes all covariates.

Statistical significance was determined if the respective statistic had a *p*-value less than 0.05. We checked for multicollinearity in the regression models and found the mean value of the variance inflation factor (VIF) to be less than 1.5. Therefore, multicollinearity is negligible in our statistical models [29]. All statistical estimations were performed using the STATA software version 16.0 [30].

## Results

### Sample description

Table 1 shows the characteristics of 8,398 children aged under five years during BDHS 2017–18. Nearly 21% of children were in the age group less than 1 year, and 52% of children were male. Nearly 7% and 15% of children had a mother and father with no formal schooling, respectively. One in every ten children come from a household with poor sanitation condition, and 89% of children are from households where drinking water is untreated. Handwashing in Public spaces was common (64%), and most of the children come from households that did not share toilets with other households (67%). Nearly 44% of the population belonged to the lowest 40% wealth quintile households. In the community context, 65% resided in rural areas, and 31% resided in high altitude communities. Further, 35% and 33% of children were from communities with low socioeconomic status and had a low maternal education level, respectively. In terms of population numeric, Chittagong is the largest division (17%), followed by the Dhaka division (15%), which includes the country’s capital city Dhaka.

**Table 1:** Absolute (N) and percentage (%) distribution of children under five years by child-level, parent-related, household-level and community-level characteristics

Characteristics	Total population	
	**N**	**%**
**Age of child (in years)**		
Four	1,694	20.2
Three	1,587	18.9
Two	1,655	19.7
One	1,666	19.8
Less than one year	1,796	21.4
**Gender of child**		
Female	4,027	48.0
Male	4,371	52.0
**Number of children under mother**		
One	6,299	75.0
Two	1,963	23.4
Three or more	136	1.6
**Mother’s level of education**		
Secondary and above	5,371	64.0
Upto primary	2,420	28.8
No formal education	607	7.2
**Father’s level of education**		
Secondary and above	4,356	51.9
Upto primary	2,811	33.5
No formal education	1,231	14.7
**Household sanitation condition**		
Poor	931	11.1
Average	3,061	36.4
Good	4,406	52.5
**Water treated before drinking**		
Yes	926	11.0
No	7,472	89.0
**Type of handwashing place**		
Private space	2,726	32.5
Public space	5,374	64.0
No handwashing place	298	3.5
**Shares Toilet with other households**		
Not shared	5,624	67.0
Shared by two HH	1,311	15.6
Shared by three HH	690	8.2
Shared by four and more HH	773	9.2
**Household wealth quintile**		
Richest	1,641	19.5
Rich	1,646	19.6
Middle	1,438	17.1
Poor	1,400	16.7
Poorest	2,273	27.1
**Religion of household**		
Islam	7,694	91.6
Hinduism	655	7.8
Others	49	0.6
**Type of season**		
Summer	662	7.9
Winter	7,233	86.1
Monsoon	503	6.0
**Type of community**		
City corporation	776	9.2
Other urban areas	2,152	25.6
Rural areas	5,470	65.1
**Altitude level of community**		
Low	3,336	39.7
Medium	2,454	29.2
High	2,608	31.1
**Socioeconomic status of community**		
Low	2,963	35.3
Medium	2,931	34.9
High	2,504	29.8
**Level of maternal education in community**		
Low	2,800	33.3
Medium	2,819	33.6
High	2,779	33.1
**Administrative division of community**		
Dhaka	1,246	14.8
Chittagong	1,393	16.6
Barisal	863	10.3
Khulna	872	10.4
Mymensingh	991	11.8
Rajshahi	874	10.4
Rangpur	934	11.1
Sylhet	1,225	14.6
**Overall**	**8,398**	**100**

Table 2 provides the morbidity profile of under-five children during 2017–18. We found that one in every three children experienced fever or cough two weeks before the survey. Moreover, 13% and 5% of children experienced respiratory infection and diarrhoea, respectively. Roughly 28% of the children experienced two or more conditions within 14 days preceding the survey.

**Table 2:** Morbidity profile of under-five children in Bangladesh 2017–18

Disease characteristics	Total population	
	**N**	**%**
**Had fever in two weeks**		
No	5,634	67.1
Yes	2,764	32.9
**Had cough in two weeks**		
No	5,357	63.8
Yes	3,041	36.2
**Acute Respiratory Infection in two weeks**		
No	7,344	87.4
Yes	1,054	12.6
**Had diarrhoea in two weeks**		
No	7,986	95.1
Yes	412	4.9
**Multimorbidity status**		
No condition	4,448	53.0
Single condition	1,586	18.9
Two conditions	1,492	17.8
Three conditions	787	9.4
Four conditions	85	1.0
**Overall**	**8,398**	**100**

### Bivariate analysis

Table 3 shows the bivariate association between morbidity incidence and the explanatory variables. Morbidity condition was higher in children aged one year than those belonging to other age categories (Single morbidity: 21%; Multimorbidity: 35%). Approximately 30% of male children experienced multimorbidity compared to 26% in females. Nearly 29% of children who drink untreated water experienced multimorbidity compared to 23% of children who drink treated water. Moreover, the incidence of multimorbidity was higher if households did not practice handwashing (32%). Additionally, the incidence of multimorbidity among children was higher during the monsoon season (31%) and in the rural areas (29%). Coming to the community characteristics, we observed that children living in high-altitude communities (31%), communities with low socioeconomic status (30%) and low maternal education (30%) had a higher incidence of multimorbidity. Additionally, multimorbidity incidence ranged from more than 31% in the Barisal, Rajshahi, and Rangpur divisions to lower than 25% in the Khulna and Dhaka divisions.

**Table 3:** Bivariate association between morbidity incidence and the child-level, parent-related, household-level and community-level characteristics

Characteristics	Total	Multimorbidity status^a^						χ2 tests of association
	**population**	**No condition**		**Single condition**		**Multiple conditions**		
	**N**	**N**	**%**	**N**	**%**	**N**	**%**	
**Age of child (in years)**								
Four	1,694	1,018	60.1	312	18.4	364	21.5	χ2 = 130.25; *p*-value = 0.001
Three	1,587	931	58.7	265	16.7	391	24.6	
Two	1,655	867	52.4	315	19.0	473	28.6	
One	1,666	729	43.8	356	21.4	581	34.9	
Less than one year	1,796	903	50.3	338	18.8	555	30.9	
**Gender of child**								
Female	4,027	2,204	54.7	758	18.8	1,065	26.4	χ2 = 12.54; *p*-value = 0.002
Male	4,371	2,244	51.3	828	18.9	1,299	29.7	
**Number of children under mother**								
One	6,299	3,241	51.5	1,234	19.6	1,824	29.0	χ2 = 26.00; *p*-value = 0.001
Two	1,963	1,120	57.1	331	16.9	512	26.1	
Three or more	136	87	64.0	21	15.4	28	20.6	
**Mother’s level of education**								
Secondary and above	5,371	2,798	52.1	1,086	20.2	1,487	27.7	χ2 = 19.18; *p*-value = 0.001
Upto primary	2,420	1,305	53.9	402	16.6	713	29.5	
No formal education	607	345	56.8	98	16.1	164	27.0	
**Father’s level of education**								
Secondary and above	4,356	2,326	53.4	820	18.8	1,210	27.8	χ2 = 5.67; *p*-value = 0.225
Upto primary	2,811	1,452	51.7	557	19.8	802	28.5	
No formal education	1,231	670	54.4	209	17.0	352	28.6	
**Household sanitation condition**								
Poor	931	526	56.5	164	17.6	241	25.9	χ2 = 8.09; *p*-value = 0.088
Average	3,061	1,593	52.0	567	18.5	901	29.4	
Good	4,406	2,329	52.9	855	19.4	1,222	27.7	
**Water treated before drinking**								
Yes	926	563	60.8	146	15.8	217	23.4	χ2 = 25.64; *p*-value = 0.000
No	7,472	3,885	52.0	1,440	19.3	2,147	28.7	
**Type of handwashing place**								
Private space	2,726	1,437	52.7	515	18.9	774	28.4	χ2 = 3.26; *p*-value = 0.515
Public space	5,374	2,858	53.2	1,022	19.0	1,494	27.8	
No handwashing place	298	153	51.3	49	16.4	96	32.2	
**Shares Toilet with other households**								
Not shared	5,624	3,011	53.5	1,053	18.7	1,560	27.7	χ2 = 9.87; *p*-value = 0.130
Shared by two HH	1,311	657	50.1	256	19.5	398	30.4	
Shared by three HH	690	348	50.4	137	19.9	205	29.7	
Shared by four and more HH	773	432	55.9	140	18.1	201	26.0	
**Household wealth quintile**								
Richest	1,641	928	56.6	314	19.1	399	24.3	χ2 = 20.92; *p*-value = 0.007
Rich	1,646	859	52.2	304	18.5	483	29.3	
Middle	1,438	744	51.7	282	19.6	412	28.7	
Poor	1,400	741	52.9	277	19.8	382	27.3	
Poorest	2,273	1,176	51.7	409	18.0	688	30.3	
**Religion of household**								
Islam	7,694	4,042	52.5	1,459	19.0	2,193	28.5	χ2 = 14.84; *p*-value = 0.005
Hinduism	655	369	56.3	120	18.3	166	25.3	
Others	49	37	75.5	7	14.3	5	10.2	
**Type of season**								
Summer	662	379	57.3	112	16.9	171	25.8	χ2 = 8.38; *p*-value = 0.079
Winter	7,233	3,823	52.9	1,373	19.0	2,037	28.2	
Monsoon	503	246	48.9	101	20.1	156	31.0	
**Type of community**								
City corporation	776	484	62.4	140	18.0	152	19.6	χ2 = 36.97; *p*-value = 0.000
Other urban areas	2,152	1,116	51.9	413	19.2	623	28.9	
Rural areas	5,470	2,848	52.1	1,033	18.9	1,589	29.0	
**Altitude level of community**								
Low	3,336	1,835	55.0	619	18.6	882	26.4	χ2 = 17.31; *p*-value = 0.002
Medium	2,454	1,292	52.6	485	19.8	677	27.6	
High	2,608	1,321	50.7	482	18.5	805	30.9	
**Socioeconomic status of community**								
Low	2,963	1,552	52.4	536	18.1	875	29.5	χ2 = 9.02; *p*-value = 0.061
Medium	2,931	1,526	52.1	574	19.6	831	28.4	
High	2,504	1,370	54.7	476	19.0	658	26.3	
**Level of maternal education in community**								
Low	2,800	1,521	54.3	454	16.2	825	29.5	χ2 = 25.64; *p*-value = 0.000
Medium	2,819	1,487	52.7	536	19.0	796	28.2	
High	2,779	1,440	51.8	596	21.4	743	26.7	
**Administrative division of community**								
Dhaka	1,246	702	56.3	236	18.9	308	24.7	χ2 = 58.53; *p*-value = 0.000
Chittagong	1,393	783	56.2	233	16.7	377	27.1	
Barisal	863	425	49.2	170	19.7	268	31.1	
Khulna	872	459	52.6	207	23.7	206	23.6	
Mymensingh	991	508	51.3	198	20.0	285	28.8	
Rajshahi	874	423	48.4	178	20.4	273	31.2	
Rangpur	934	470	50.3	171	18.3	293	31.4	
Sylhet	1,225	678	55.3	193	15.8	354	28.9	
**Overall**	**8,398**	**4,448**	**53**	**1,586**	**19**	**2,364**	**28**	

^a^For ease of dissemination, Multimorbidity status of children is categorized into No condition, Single condition, and Multiple conditions

### Multivariate analysis

Table 4 shows the community-level random-effects measures and model fit statistics from the two-level random intercept Poisson regression models. We observe that the community-level variability across all the models is statistically significant at the 5% level. Moreover, on average, the incidence rate of multimorbidity is 1.34 times higher among children from high-risk communities in the null model compared to low-risk communities. Further, the community-level variance and heterogeneity in the risk of multimorbidity decrease after including the child, parent and household-related covariates (model-I) and decreases further after inclusion of community-level covariates (model-II). Additionally, all models’ statistically significant likelihood ratio tests imply that the two-level Poisson regression model fits better than a standard Poisson regression model.

**Table 4:** Community-level random effects and model characteristics of the random intercept Poisson regression models of the risk of multimorbidity among under-five Bangladeshi children

Random-effects measures	Multimorbidity status^a^		
	**Null Model**^**b**^	**Model-I**^**b**^	**Model-II**^**b**^
**Level 2: Community**			
Variance	0.096	0.081	0.072
Variance 95% Confidence Interval (CI)	(0.076, 0.121)	(0.063, 0.104)	(0.055, 0.094)
Median Rate Ratio (MRR)	1.34	1.31	1.29
**Likelihood Ratio Test statistic**^**c**^	194.81	149.48	126.72
**Likelihood Ratio Test** ***p*-value**	0.001	0.001	0.001
**No of communities**	672	672	672
**No of children**	8,398	8,398	8,398

^a^ Multimorbidity status is a count variable of the number of conditions a child suffered from in two weeks preceding the survey; ^b^ Null model is an empty model without any covariates, Model-I includes all covariates excluding the community-level characteristics, Model-II includes all covariates; ^c^ Likelihood ratio tests were performed against standard Poisson regression models with the same covariates respectively

Table 5 gives the multivariate association of multimorbidity status and the explanatory variables. The full model shows that children aged less than one year were 1.41 times [95% CI: 1.30, 1.52] more likely to experience multimorbidity than children aged four years. Moreover, the likelihood of multimorbidity was 1.11 times [CI: 1.06, 1.17] higher among male children compared to their female counterparts. Children from the poorest wealth quintile household were 1.16 times [CI: 1.04, 1.29] more likely to experience multimorbidity. Further, the incidence of multimorbidity was 1.31 times [CI: 1.07, 1.60] times higher during the monsoons corresponding to the summer season. According to the community characteristics, children residing in rural areas and other urban areas involved 1.29 [CI: 1.11, 1.51] and 1.28 [CI: 1.11, 1.47] times greater risk of multimorbidity than children from city corporations. Further, children from medium- and high-altitude communities had 1.11 [CI: 1.01, 1.22] and 1.16 [CI: 1.03, 1.30] times greater risk of multimorbidity compared to children living in low-altitude communities. In contrast to the bivariate results, the multivariate association of socioeconomic status and maternal education in a community with multimorbidity status was not statistically significant at the 5% level. Comparing the information criterion and the statistically significant likelihood-ratio test, it is evident that the model with community characteristics better predicts the incidence of multimorbidity compared to the model without any community-level covariates.

**Table 5:** Incidence rate ratio from multilevel Poisson models showing the multivariate association between morbidity incidence and the child-level, parent-related, household-level and community-level characteristics

Fixed-effects characteristics	Morbidity status^e^			
	**Model-I**^**f**^		**Model-II**^**f**^	
	**IRR**^**a**^	**95% CI**^**b**^	**IRR**^**a**^	**95% CI**^**b**^
**Age of child (in years)**				
Four	Ref		Ref	
Three	1.08*	(0.99—1.17)	1.08*	(0.99—1.17)
Two	1.28***	(1.18—1.38)	1.28***	(1.18—1.38)
One	1.55***	(1.44—1.67)	1.55***	(1.44—1.67)
Less than one year	1.41***	(1.30—1.52)	1.41***	(1.30—1.52)
**Gender of child**				
Female	Ref		Ref	
Male	1.12***	(1.07—1.17)	1.11***	(1.06—1.17)
**Number of children under mother**				
One	Ref		Ref	
Two	0.90***	(0.85—0.96)	0.91***	(0.85—0.96)
Three or more	0.75***	(0.60—0.93)	0.74***	(0.60—0.92)
**Mother’s level of education**				
Secondary and above	Ref		Ref	
Upto primary	1.00	(0.94—1.06)	1.01	(0.95—1.07)
No formal education	0.92	(0.83—1.03)	0.94	(0.84—1.04)
**Father’s level of education**				
Secondary and above	Ref		Ref	
Upto primary	1.01	(0.96—1.08)	1.02	(0.96—1.08)
No formal education	0.98	(0.90—1.06)	0.97	(0.90—1.06)
**Household sanitation condition**				
Poor	Ref		Ref	
Average	1.10**	(1.01—1.20)	1.11**	(1.02—1.21)
Good	1.07	(0.98—1.17)	1.07	(0.98—1.17)
**Water treated before drinking**				
Yes	Ref		Ref	
No	1.11**	(1.01—1.22)	1.03	(0.93—1.14)
**Type of handwashing place**				
Private space	Ref		Ref	
Public space	0.92***	(0.87—0.98)	0.92**	(0.87—0.98)
No handwashing place	1.05	(0.92—1.21)	1.05	(0.91—1.20)
**Shares Toilet with other households**				
Not shared	Ref		Ref	
Shared by two HH	1.05	(0.99—1.13)	1.06	(0.99—1.13)
Shared by three HH	1.08*	(0.99—1.18)	1.08*	(0.99—1.18)
Shared by four and more HH	0.96	(0.87—1.05)	1.00	(0.91—1.09)
**Household wealth quintile**				
Richest	Ref		Ref	
Rich	1.18***	(1.08—1.28)	1.14***	(1.05—1.25)
Middle	1.15***	(1.05—1.26)	1.11**	(1.00—1.22)
Poor	1.15***	(1.04—1.27)	1.09*	(0.98—1.22)
Poorest	1.22***	(1.11—1.34)	1.16***	(1.04—1.29)
**Religion of household**				
Islam	Ref		Ref	
Hinduism	0.92*	(0.83—1.02)	0.91*	(0.83—1.01)
Others	0.48***	(0.29—0.77)	0.47***	(0.29—0.76)
**Type of season**				
Summer	Ref		Ref	
Winter	1.12*	(0.99—1.27)	1.05	(0.92—1.20)
Monsoon	1.21**	(1.02—1.45)	1.31***	(1.07—1.60)
**Type of community**				
City corporation			Ref	
Other urban areas			1.28***	(1.11—1.47)
Rural areas			1.29***	(1.11—1.51)
**Altitude level of community**				
Low			Ref	
Medium			1.11**	(1.01—1.22)
High			1.16**	(1.03—1.30)
**Socioeconomic status of community**				
Low			Ref	
Medium			1.00	(0.92—1.09)
High			1.05	(0.93—1.18)
**Level of maternal education in community**				
Low			Ref	
Medium			0.98	(0.90—1.07)
High			0.98	(0.89—1.08)
**Administrative division of community**				
Dhaka			Ref	
Chittagong			1.21**	(1.04—1.40)
Barisal			1.32***	(1.12—1.56)
Khulna			1.16*	(0.99—1.36)
Mymensingh			1.13	(0.96—1.32)
Rajshahi			1.23**	(1.04—1.45)
Rangpur			1.15	(0.96—1.38)
Sylhet			1.16*	(0.99—1.36)
**Degrees of freedom**	29		44	
**Akaike’s information criterion (AIC)**	21,631.59		21,620.15	
**Bayesian information criterion (BIC)**	21,835.63		21,929.72	
**Likelihood-ratio test between Model I and II**			41.44***	
**Number of communities**	672		672	
**Number of children**	8,398		8,398	

^a^ *IRR* incidence rate ratio; ^b^ 95% Confidence Interval (CI) is given in brackets; ^c^ Statistical significance is denoted by asterisks where *** *p*-value < 0.01, ** *p*-value < 0.05, * *p*-value < 0.1; ^d^ Ref. denotes reference category; ^e^ Multimorbidity status is a count variable of the number of conditions a child suffered from in two weeks preceding the survey; ^f^ Model-I includes all covariates excluding the community-level characteristics, Model-II includes all covariates

## Discussion

Although significant progress has been made to curb the spread of infectious diseases in Bangladesh, the present study shows that more than one morbid condition in under-five children is high. The bivariate association showed that some of the child, parent, and household-level characteristics are significantly associated with multimorbidity of infectious diseases in under-five Bangladeshi children. Moreover, an influential role of community was observed in the presence of multiple infectious diseases in children while performing the multilevel Poisson regression model. This finding is supported by significant between-community variance in multimorbidity of infectious diseases after adjusting the contextual level factors. The type of community, the altitude of the community, and the administrative divisions of the community were consistent predictors of the multimorbidity status.

Empirical evidence shows that child’s age influences the multimorbidity of infectious diseases. While comparing different age groups, children in their first year of life were expected to have a higher rate of multiple infectious diseases. This is not surprising since it has been usually found that children at their younger ages can get exposed to contaminated water, soil, and food easily. At these ages, they usually crawl and try to explore the environment. These results were consistent with other studies, showing that the younger children had higher odds of diarrhoea and acute respiratory infection along with fever and cough [13, 31]. Moreover, higher age group children who have already moved towards the environment exposure are well-versed and sometimes build a strong immunity till that age, and show lesser prevalence in infectious diseases. While previous studies have usually stated that male children are more preferred towards food allocation and health care availability in a household, making them lesser prone to caught infectious diseases [21]. In contrast, the present study found that the rate of multiple infectious diseases was higher among male children than their female counterparts. One possible mechanism through which such an effect can operate has been shown in an Indian study where a lesser chance of neonatal mortality was observed among female children, indicating the importance of the biological capacity of female children in initial ages [32].

Although household and environmental factors had an influential role in the prevalence of infectious diseases in children, the present study found that the children residing in a household with poor sanitation conditions experience a lesser rate of multiple infectious diseases than those with average facilities. This result is consistent with a previous study, indicating the excellent health outcomes paradox despite economic deprivation in Bangladesh [33, 34]. Even in the case of consuming treated drinking water, we did not find any significant association with multiple infectious diseases after adjusting the community-level characteristics. While the quality of water is an essential predictor of childhood illnesses, in some cases, deprivation from basic facilities like quantity and convenient water supply plays an efficient role [35, 36]. Some areas in Bangladesh still struggle to have proper accessibility of water supply, explaining the higher rate of multiple infectious diseases in households without water. We found a significant association of public handwashing space with a lower rate of multimorbidity of infectious diseases in under-five children. Since sometimes, it’s not just the handwashing station that makes the difference, but the handwash practising behaviour of people in a community may inspire others.

Consistent with previous studies, this study shows that children from the wealthiest wealth quintile household had a lesser rate of experiencing multimorbidity of infectious diseases than those with poorest quintile households [13, 37]. Moreover, scarce literature shows that affluent wealth quintile households may face multimorbidity problems of infectious diseases in children equivalent to the poorest quintile households, which can also be observed in the present study [38]. Multiple morbidities of infectious diseases were significantly higher during monsoon seasons which is consistent with a previous study showing the health impact of climate change [39]. Our study shows that children residing in rural and urban areas that are not cities (i.e., other urban areas) reported a higher rate of multimorbidity in infectious diseases. One of the plausible reasons for such association may be exposure to an unhealthy environment in rural areas. Those urban areas surrounded by such an environment may also face a disproportionate burden of poor health [40].

Additionally, the higher and medium-altitude of the community also affects the multimorbidity of infectious diseases in under-five children. Barisal administrative division followed by Rajshahi and Chittagong shows the highest rate of multimorbidity in infectious diseases. This may be due to the higher indigenous population in the Chittagong area who are yet deprived of basic facilities. Moreover, a WHO report has shown that poverty in the administrative divisions like Rajshahi and Barisal had increased throughout the decade resulted in a weak health care system [41].

Studies carried out in Bangladesh have primarily focused on diarrhoea and acute respiratory tract infections independently without considering their co-occurrence effect. And such studies have further led to bringing interventions separately for these infectious diseases. Using Bangladesh’s large-scale, nationally representative data, the present study adds to the knowledge of the coexistence of childhood infectious diseases and their determinants. Moreover, this study provides evidence of community-level factors being associated with the coexistence of diarrhoea and acute respiratory infections in under-five Bangladeshi children. Despite such advantages, the limitations of this study must also be noted. The cross-sectional nature of data does not allow us to draw any causal inferences. The information of morbidity incidence that was self-reported by the mother may suffer from recall bias. However, the short recall period of morbidity (two weeks before the survey) makes this chance minimal.

## Conclusion

The present study brings forward the growing multimorbidity issue of infectious diseases among under-five children in Bangladesh. It provides evidence of the influence of community-level factors on the coexistence of diarrhoea and acute respiratory infections. The significant effect of public handwashing places suggests focussing on community-based interventions in which individuals learn and promote hygiene habits among themselves, eventually reducing the prevalence of co-existing infectious diseases. A higher incidence of coexistence of such infectious diseases in the poor and semi-urban populace further highlights the environmental effect among individuals regardless of their economic status. The significant effect of altitude of community recommends implementing risk reduction programs in high-risk areas where higher coexistence of diarrhoea and acute respiratory infections exists among under-five children. The government should take advantage of shared characteristics and bonds among the community. Policymakers should focus on community-level awareness and programs to strengthen the primary health care system. Growing multimorbidity cases among under-five children in higher altitude areas, monsoon-affected regions, divisions with a higher indigenous population, and a weaker health system indicate the need for targeted health policies to tackle the simultaneous occurrence of infectious diseases across the high-risk pockets in Bangladesh.

## Data Availability

The study utilizes a secondary source of data that is freely available in the public domain through The DHS Program—Bangladesh: Standard DHS, 2017–18 .

## Abbreviations

BDHS: Bangladesh Demographic and Health Survey
NIPORT: National Institute of Population Research and Training
ARI: Acute Respiratory Infections
HH: Households
IRR: Incidence Rate Ratios
MRR: Median Rate Ratios
CI: Confidence Interval
